# Leveraging genomic signatures of oral microbiome-associated antibiotic resistance genes for diagnosing pancreatic cancer

**DOI:** 10.1371/journal.pone.0302361

**Published:** 2024-04-30

**Authors:** Xiaojing Shen, Xiaolin Zhu, Hairong Liu, Rongtao Yuan, Qingyuan Guo, Peng Zhao

**Affiliations:** 1 Qingdao municipal hospital(group) Stomatology, Qingdao, Shandong, China; 2 Department of Gastroenterology, Qingdao Municipal Hospital, Qingdao, Shandong, China; Penn State Health Milton S Hershey Medical Center, UNITED STATES

## Abstract

Growing evidence has increasingly suggested a potential linkage between the oral microbiome and various diseases, including pancreatic ductal adenocarcinoma (PDAC). However, the utilization of gene-level information derived from the oral microbiome for diagnosing PDAC remains unexplored. In this study, we sought to investigate the novel potential of leveraging genomic signatures associated with antibiotic resistance genes (ARGs) within the oral microbiome for the diagnosis of PDAC. By conducting an analysis of oral microbiome samples obtained from PDAC patients, we successfully identified specific ARGs that displayed distinct sequence abundance profiles correlated with the presence of PDAC. In the healthy group, three ARGs were found to be enriched, whereas 21 ARGs were enriched in PDAC patients. Remarkably, these ARGs from oral microbiome exhibited promising diagnostic capabilities for PDAC (AUROC = 0.79), providing a non-invasive and early detection method. Our findings not only provide novel modal data for diagnosing PDAC but also shed light on the intricate interplay between the oral microbiome and PDAC.

## Introduction

Over the past decade, advancements in high-throughput sequencing technologies, particularly metagenomics, have revolutionized our understanding of the oral microbiome [1–3]. These techniques allow researchers to identify and analyze the vast array of microorganisms present in the oral cavity, beyond those that can be cultivated using traditional microbiological methods. The oral microbiota is a complex ecosystem composed of bacteria, fungi, viruses, and archaea, all working in harmony or dysbiosis depending on the balance of their populations [4]. Understanding the composition, diversity, and functionality of oral microbiota is essential for host health [5, 6]. Notably, the oral microbiota has been associated with systemic health conditions [7, 8].

The human microbiome contains repositories of antibiotic resistance genes (ARGs) that are present across numerous microbiomes. Surveillance of ARGs is a key tenet of the human health [9]. The oral microbiome serves as a recognized hub for acquiring and disseminating ARGs, playing a role in the widespread emergence of antimicrobial-resistant infections [10]. Previous has described the oral microbiome diversity and prevalence of ARGs in periodontal health and disease [11]. Specifically, recent studies have shed light on the intricate relationship between the oral microbiome and pancreatic cancer (PDAC) [12], a highly aggressive malignancy with a low survival rate. Furthermore, the oral microbiome exhibits the potential to serve as a source of biomarkers for the early detection and diagnostic stratification of PDAC [12]. Specific microbial signatures and changes in microbial gene expression have been identified in PDAC patients, suggesting their potential utility as non-invasive diagnostic tools. However, there are additional biomarkers, specifically at the gene level, that have yet to be explored, such as ARGs. ARGs play a crucial role in microbial populations and have gained significant attention in recent years due to their impact on human health [11, 13], including their association with various diseases and their potential as diagnostic markers [14]. The presence of ARGs within the oral microbiome can provide insights into the microbial ecology, as well as antibiotic usage and exposure history of an individual [15, 16].

One study by Fan et.al observed that patients with PDAC were more likely to have antibodies against *Porphyromonas gingivalis*, an oral bacterium, indicating a past infection [17]. The presence of pathogenic bacteria has been proposed to contribute to systemic inflammation, which is a recognized risk factor for the development of PDAC. Further, Fulop et.al found that receipt of perichemotherapy antibiotics was associated with improved survival among patients treated with gemcitabine [18]. They think that antibiotics may modulate bacteria-mediated gemcitabine resistance and have the potential to improve PDAC outcomes. In the context of PDAC, understanding the abundance and diversity of ARGs within the oral microbiome could have important implications.

In this study, our primary objective was to explore a novel approach by examining the untapped potential of utilizing genomic signatures specifically linked to ARGs present within the saliva for improving the diagnostic capabilities of PDAC. By focusing on these genomic signatures, we aimed to investigate their association with PDAC and evaluate their effectiveness in distinguishing between individuals with PDAC and those without, offer a non-invasive and promising avenue for improving patient outcomes.

## Materials and methods

### Data collection

**Fig 1:**
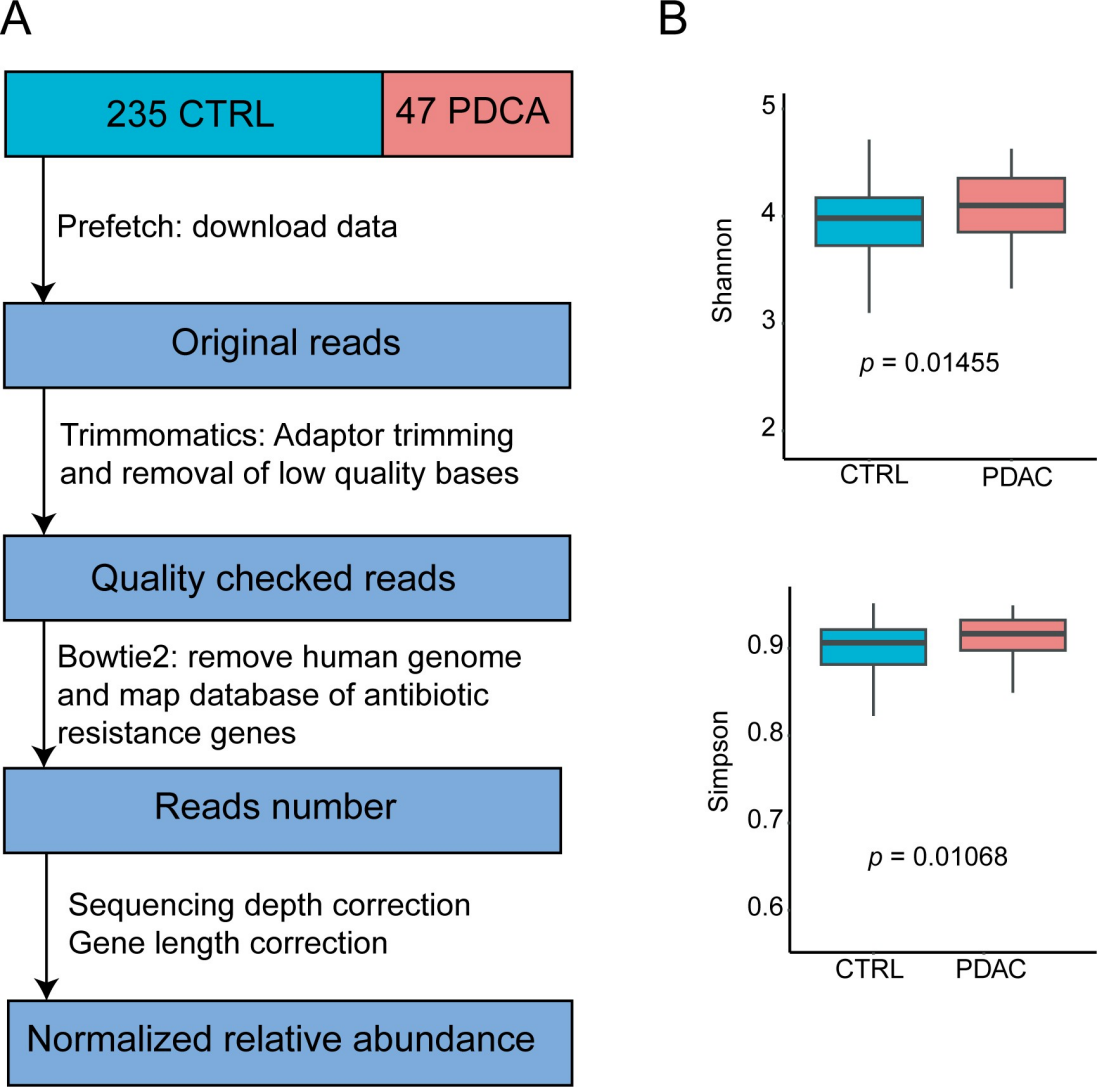
The analysis workflow and the calculation of alpha diversity. (A) Data acquisition and processing workflow. (B) Calculation and comparison of alpha diversity using Shannon and Simpson indices.

47 salivary samples from patients with PDAC and 235 salivary from healthy control were collected by Nagata et.al study [12]. Notably, the authors considered confounding factors such as gender and age when collecting saliva samples from these patients and healthy control. Furthermore, these confounding factors does not affect the composition of the oral microbiome. Therefore, in this study, we no longer consider the influence of confounding factors. The data was downloaded using the prefetch v2.10.7 (https://github.com/ncbi/sra-tools). The process of data collection and processing can be found in Fig 1A.

### Quality control of shotgun metagenomic sequence

Subsequently, Trimmomatic v0.39 [19] was employed to remove adapter sequences and low-quality bases (ILLUMINACLIP:TruSeq3-PE-2.fa:2:30:10:8:true TRAILING:20 MINLEN:60). After quality control, the reads were further processed to remove human genomic sequences using bowtie v2.4.4 [20]. To achieve this, we utilized the T2T-mY-rCRS genome [21] (https://github.com/marbl/CHM13, PARs on chrY hard masked to "N" and mitochondrion replaced with rCRS), which is essential for effectively removing human genomic contamination, particularly in saliva samples with high host genomic content.

### ARGs annotation

Following the removal of human genomic sequences, we performed alignments against the Comprehensive Antibiotic Resistance Database (CARD [22], https://card.mcmaster.ca/download) using bowtie v2.4.4 [20] and samtools v1.13 [23]. This enabled us to determine the number of reads that matched ARGs in each sample. To accurately quantify their abundance, we applied corrections for both sequencing depth and the gene lengths specific to the ARGs [14]. Consequently, standardized relative abundance information was obtained, which will be utilized for subsequent analyses.

### Diagnostic model construction

A Random Forest classifier was utilized in this study to construct a diagnostic model for pancreatic cancer. Our research dataset consists of genomic features related to the oral microbiome, specifically antibiotic resistance genes, with the aim of utilizing them fully for diagnosing pancreatic cancer. Initially, the dataset is loaded. The dataset contains information regarding various genomic features and their respective disease outcomes. The dataset is divided into input features (X) and target variables (y). X encompasses the genomic features, while y represents the disease outcomes. Subsequently, highly correlated features with the disease outcome are filtered out by calculating Pearson correlation coefficients between each feature and the target variable. The top 2000 most relevant features were selected for further analysis. Then, the dataset is split into a training set and a test set, allocating 80% of the data for training and 20% for testing. Lastly, a diagnostic model is built using a Random Forest classifier. Parameters such as the number of decision trees (n_estimators) and the maximum depth of trees (max_depth) are configured to optimize performance on the test set. The performance of the model is evaluated by calculating ROC curves and AUC values (area under the curve). These metrics aid in measuring the classification performance of the model.

### Statistical analysis

The statistical analyses were conducted using R 4.3.2. Alpha diversity (Shannon and Simpson) was calculated by vegan package. To calculate the Bray-Curtis distance, the ARGs profiles was directly computed. PCoA analysis was performed in R using the ade4 package [24]. The Adonis analysis was conducted using the vegan package. To test the differential abundances of ARGs, the Wilcoxon rank-sum test was employed, and the Benjamini-Hochberg (BH) procedure was used to correct the p-values, with significance defined as p-adjust < 0.05. For creating boxplots and PCOA plot, the ggplot2 package was utilized. The heatmap was constructed using the pheatmap package. The receiver operating characteristic (ROC) curves were built using the pROC package. Additionally, bioinformatic analysis was performed using the OmicStudio tools athttps://www.omicstudio.cn/tool.

## Results

### PDAC patients have higher alpha diversity of ARGs in their oral microbiome

We determined the normalized sequence relative abundance of ARGs in all samples through the analysis workflow shown in Fig 1A. To explain the distribution of oral ARGs in PDAC patients, in Table 1, we present the top 20 ARGs with the highest average relative abundance in each group and across all samples, listed as tet(M), mel, tetA(46), ErmX, ErmB, tetB(46), RlmA(II), tet(O/M/O), tet(W/N/W), tet(W), tet(Q), patA, patB, tetA(60), CfxA, CfxA3, lsaC, pmrA, CfxA2 and ErmF. We found that the composition of ARGs in the top 20 of healthy people and PDAC patients was the same, but the rank of each ARGs was different. We found that these ARGs are mainly associated with tetracycline, erythromycin and cephalosporinase in healthy and PDAC group. The relative abundance ranking of ARGs differs in PDAC and healthy control, suggesting a potential difference in ARG composition. Subsequently, we computed the alpha diversity of ARGs encoded by the oral microbiome in PDAC patients and healthy individuals using normalized sequence relative abundance, including Shannon and Simpson diversity. We found that the ARGs of oral microbiome of PDAC patients exhibited higher alpha diversity (Fig 1B), including Shannon (p = 0.01455) and Simpson (p = 0.01068). The implications of these findings suggested a potential link between the oral microbiome composition, ARGs, and the presence of PDAC, highlighting the importance of further exploring and understanding the associations between ARGs of oral microbiome and PDAC.

**Table 1:** The abundance of top 20 ARGs in each group and across all samples.

	PDAC (%)	CTRL (%)	All (%)
ARO:3000186|tet(M)	13.18	15.33	14.25
ARO:3000616|mel	10.71	13.06	11.88
ARO:3004032|tetA(46)	7.08	8.67	7.87
ARO:3000596|ErmX	8.73	5.21	6.96
ARO:3000375|ErmB	5.53	6.95	6.24
ARO:3004033|tetB(46)	5.19	4.39	4.79
ARO:3001301|RlmA(II)	4.94	4.22	4.58
ARO:3007120|tet(O/M/O)	3.82	4.09	3.96
ARO:3004442|tet(W/N/W)	4.17	3.67	3.92
ARO:3000194|tet(W)	3.11	4.11	3.61
ARO:3000191|tet(Q)	2.31	3.83	3.07
ARO:3000024|patA	2.72	2.54	2.62
ARO:3000025|patB	2.74	2.35	2.54
ARO:3004035|tetA(60)	1.83	2.17	2.01
ARO:3003001|CfxA	1.88	1.83	1.86
ARO:3003003|CfxA3	1.91	1.72	1.81
ARO:3003112|lsaC	1.91	1.43	1.71
ARO:3000822|pmrA	1.95	1.46	1.71
ARO:3003002|CfxA2	1.44	1.88	1.66
ARO:3000498|ErmF	1.57	1.09	1.33

### ARGs with differentially abundance between PDAC and healthy control

**Fig 2:**
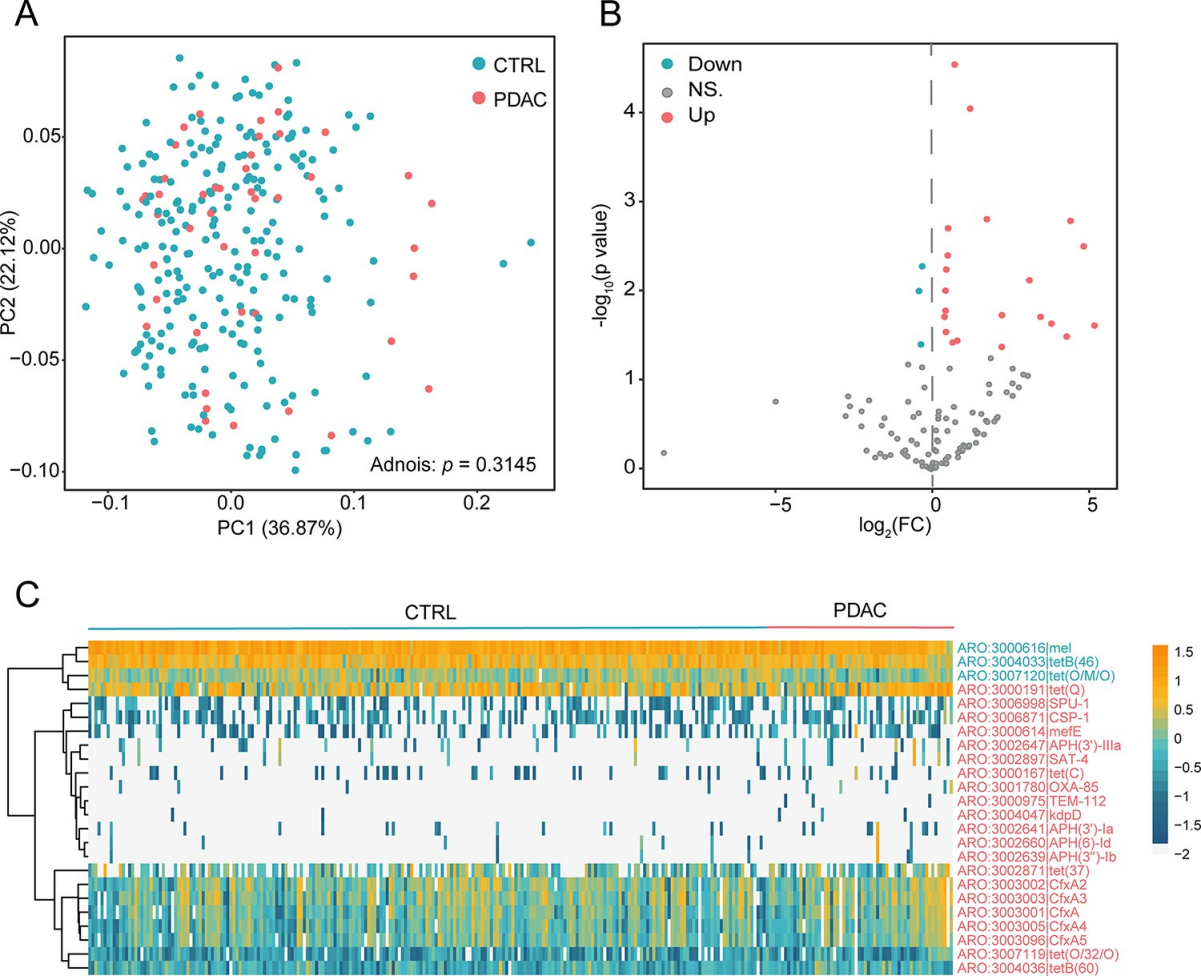
Beta diversity and differential AGRs. (A) Assessment of beta diversity based on Bray-Curtis distance and p-values calculated using Adnois. (B, C) Calculation of differentially abundant ARGs, with p-values adjusted using the Benjamini-Hochberg correction.

Subsequently, we delved into a detailed exploration of the differentiation in ARGs between individuals with PDAC and those in health by assessing beta diversity measures. Based on the relative abundance of ARGs, we calculated the Bray-Curtis distance and assessed the dissimilarity using Adonis. However, we found no significant difference in beta diversity between the two groups (Fig 2A, p = 0.3145). Undeterred by the lack of significant differences in beta diversity, we proceeded to scrutinize the distinctiveness of ARGs present in both groups. We compared the abundance differences of ARGs in PDAC and healthy individuals, with p-values adjusted by the BH adjust method. We identified a total of 24 differentially abundant ARGs (Fig 2B), with 3 enriched in healthy individuals and 21 significantly elevated in PDAC. This marked enrichment of numerous ARGs in the PDAC group suggests a potential link between antibiotic resistance and the occurrence of PDAC. To provide a comprehensive understanding of these findings, we further calculated the fold change values and visually presented them in Fig 2B. We found some ARGs that varied more than twice in abundance, suggesting that these ARGs might be biomarkers for PDAC patients. Subsequently, we presented the abundance information of these ARGs using a heatmap (Fig 2C). It can be observed that ARGs represents the mel, tetB(46) and tet(O/M/O) ARGs enriched in the healthy group, while tet(Q), APH(3’)-IIIa, SAT-4, APH(3’’)-Ib, CfxA2, CfxA4, CfxA3, CfxA5, tet(O/32/O), OXA-85, APH(3’)-Ia, tet(37), SPU-1, tetB(60), CfxA, CSP-1, TEM-112, mefE, kdpD, tet(C) and APH(6)-Id represents the 21 ARGs enriched in PDAC. Overall, we were able to identify biomarkers at the genetic level in the oral microbiome of PDAC patients that need to be explored further.

### Co-occurrence analysis showed different correlations among ARGs in healthy and PDAC group

**Fig 3:**
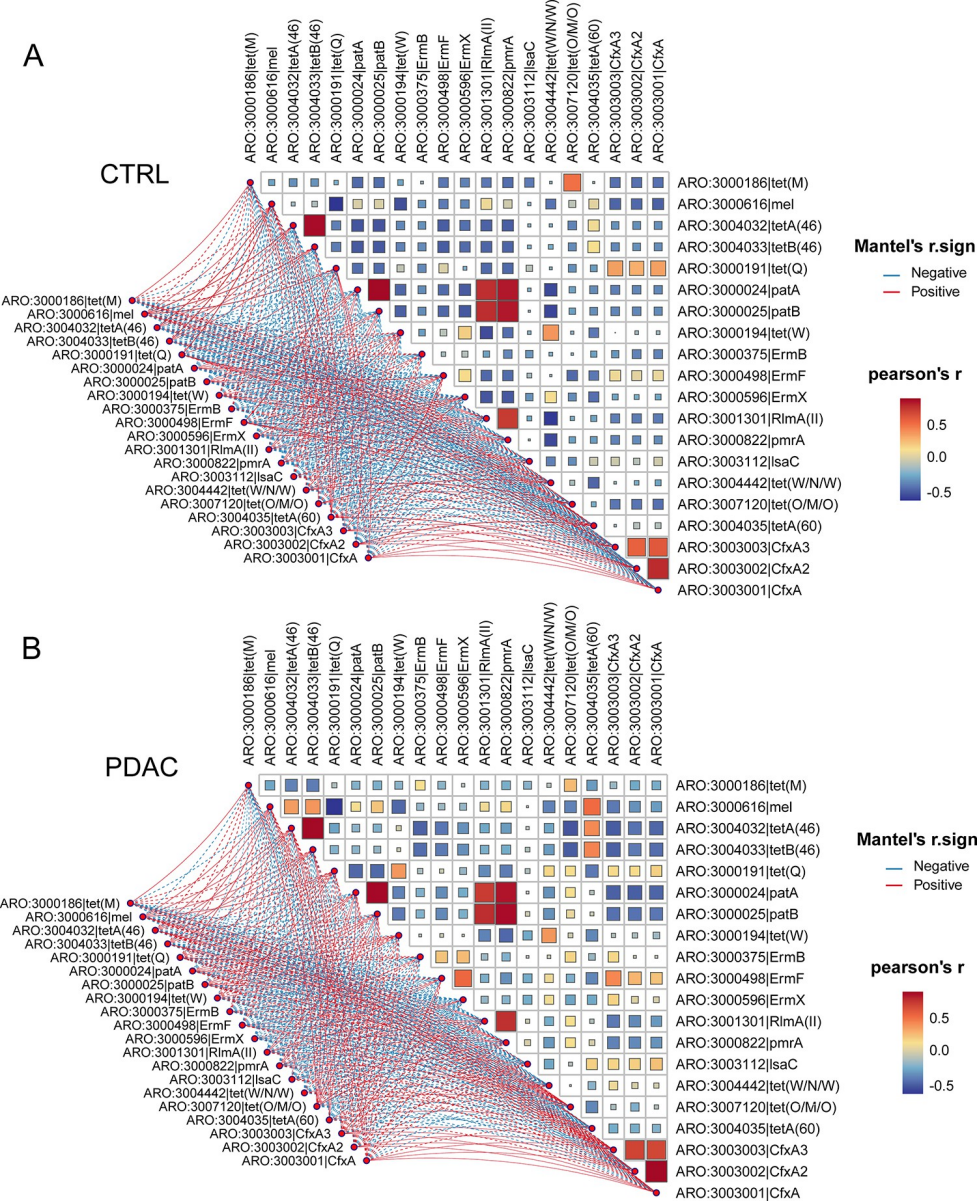
The co-occurrence correlation among ARGs. (A) Healthy group. (B) PDAC group. Pearson correlation was used in this analysis.

The exploration of co-occurrence correlations among ARGs serves as a valuable approach to unveil potential synergistic or antagonistic interactions among these genes and their impact on the host. To investigate this, we calculated the co-occurrence correlation among the top 20 abundant ARGs using Pearson correlation in both PDAC and healthy individuals (Fig 3A, 3B). By comparing the differences in co-occurrence between the two groups, we found significant co-occurrence correlations between tetA(46) and tetB(46), patA and patB, RlmA(II) and pmrAas, among CfxA3, CfxA2 and CfxA as well as among patA, patB, RlmA(II) and pmrA in both groups. However, In the healthy individual cohort, a strikingly robust co-occurrence correlation was identified between tet(W/N/W) and other ARGs, a phenomenon not replicated in the PDAC group. Conversely, in the PDAC cohort, a more robust co-occurrence correlation was observed between tetA(60), ErmF, and other ARGs, a correlation absent in the healthy individuals. This delineation of co-occurrence patterns sheds light on the intricate relationships and potential interactions among specific ARGs in the context of PDAC and healthy states.

### ARGs can be used for PDAC diagnosis

**Fig 4:**
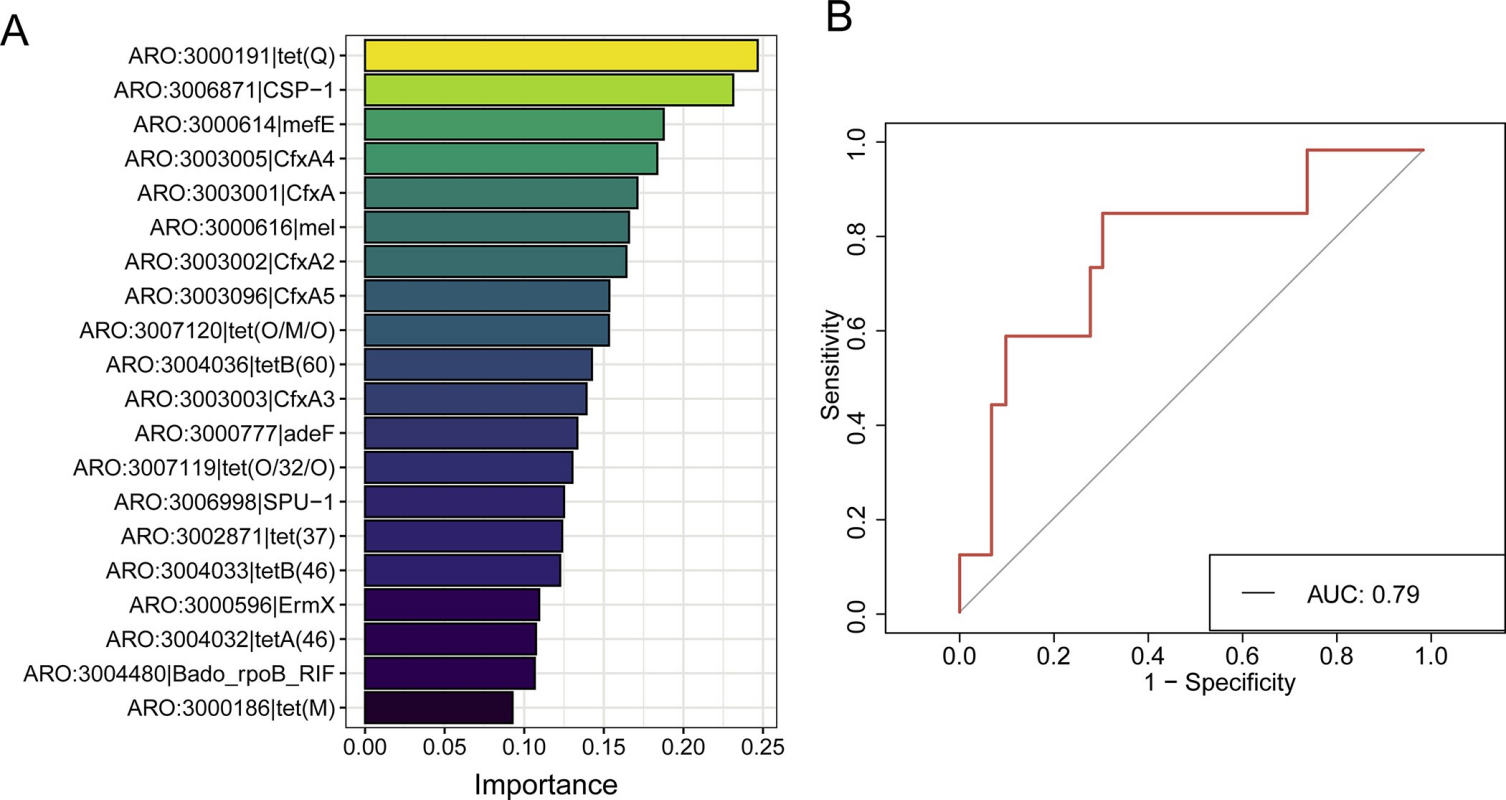
Construction of the diagnostic model. (A) Identification of the top 20 ARGs contributing the most to the model. (B) Evaluation of the accuracy of the diagnostic model.

The specific distribution of ARGs in PDAC suggests their potential use as a novel modality for diagnosing PDAC. To achieve this, we developed a predictive model for PDAC using profiles of ARGs. The implementation of the random forest algorithm demonstrated strong diagnostic abilities, exhibiting high accuracy and robustness in identifying pancreatic cancer. In Fig 4A, we present the top 20 ARGs that contributed most to the model, predominantly tet(Q), CSP-1, and mefE, which are known for their resistance against various antibiotics. Furthermore, we trained the model that the model’s diagnostic accuracy in differentiating PDAC based on these ARG profiles stood at an impressive 79%, underscoring the potential of ARGs harbored within the oral microbiome as promising alternative biomarkers for the early detection and diagnosis of PDAC.

## Discussion

The oral microbiome has gained substantial attention in recent years due to its potential role in various systemic diseases [1], including PDAC [12]. In this study, we investigated ARGs of oral microbiome in patients with PDAC. Our findings revealed significant alterations in the ARGs of oral microbiome of PDAC patients compared to healthy controls. Specifically, we observed the increase in alpha diversity, as evidenced by Shannon diversity and Simpson diversity index. The diversity of ARGs may suggest that PDAC patients have been exposed to a range of antibiotics. Due to this exposure, selecting for a more diverse set of resistant bacteria could potentially occur within their microbiomes. A higher ARG diversity within PDAC patients’ microbiome could have direct implications for treatment, as it may predispose to more frequent or severe bacterial infections that are resistant to standard antibiotics. Previous study found that oral antibiotics can activate antitumor immunity and suppress tumor burden in PDAC tumor-bearing mice [25], and clinical antibiotics can increase survival and progression-free survival of patients [18, 26]. Although antibiotics are beneficial for PDAC, the accumulation of ARGs in the human symbiotic microbiome needs to be considered as antibiotic resistance has become a significant global health concern. In addition, there exists moderate evidence indicating that the excessive or prolonged utilization of antibiotics throughout an individual’s lifespan is correlated with a slight elevation in the risk of diverse cancer [27]. Hence, understanding the prevalence of ARGs in the oral microbiome is crucial [9, 28, 29]. We identified a variety of ARGs, indicating a potential reservoir of resistance genes within the oral microbial community. Our findings highlight the importance of considering the oral microbiome as a potential source of antibiotic resistance in PDAC patients. Further understanding of the effects of these ARGs on the host is essential.

Furthermore, we identified specific ARGs that exhibited differential abundance in PDAC patients, highlighting their potential as biomarkers for the disease. These findings support previous research demonstrating the dysregulation of the oral microbiome in PDAC [12, 30]. However, PDAC is one of the most aggressive malignant tumors characterized by the lack of biomarkers for early detection [31]. To address the tissue, we have developed a novel PDAC diagnostic model with an accuracy of 79% based on ARGs of oral microbiome, Comparing the performance of the diagnostic models could provide valuable insights and contribute to improving the overall diagnostic accuracy and treatment options for PDAC patients. The accuracy of model using gut microbiota profiles based on Japanese, Spanish and German cohorts was 74% to 83% [12]. In addition, Farrell et al. integrated two microbes as bacterial biomarkers for PDAC, achieving an accuracy of 90% [32], though the biomarkers could not be confirmed [33]. However, previous studies found there is likely to be population heterogeneity in oral microbes as biomarkers [32–35]. Hence, Applying the model to cross-population cohorts is one of the means to test noninvasive diagnosis [12, 36]. Although our research has made significant progress based on functional genome rather than microbial composition, the diagnosis model needs further validation and improvement by considering populations from different countries with varying dietary habits and characteristics. Our research cannot explain the reasons for ARGs enrichment and their effects on the host, which require further investigation.

In conclusion, we revealed the distribution of ARGs of oral microbiome in PDAC patients and developed a diagnostic model for PDAC based on the functional genome of oral microbiota. Our model will further drive the exploration of multi-modal data in disease diagnosis. Further research is warranted to explore the functional implications of these findings. Understanding the interplay between the oral microbiome, PDAC, and antibiotic resistance will contribute to improved therapeutic interventions and personalized treatment strategies for patients with this devastating disease.
